# The Impact of Laparoscopic Sleeve Gastrectomy on Thyroid Functions in Egyptian Patients with Obesity

**DOI:** 10.1007/s11605-023-05662-4

**Published:** 2023-04-05

**Authors:** Mohamed Esam El-Din Mostafa Kamal, Hesham Ahmed Abou Aisha, Mohamed H. Fahmy, Amir K. Abosayed

**Affiliations:** 1 El Warraq Central Hospital, Ministry of Health, Giza, Egypt; 2 General Surgery Department, Faculty of Medicine, Cairo University, Giza, Egypt

**Keywords:** Obesity, Laparoscopic sleeve gastrectomy, Thyroid functions

## Abstract

**Background:**

Sleeve gastrectomy (SG) continues to be one of the most popular bariatric procedures all over the world. Thyroid-stimulating hormone (TSH) frequently shows a slight elevation in patients with obesity. The effect of SG on thyroid hormones has been rarely investigated.

**Aim of the Study:**

This study aimed to assess the short-term effect of SG on thyroid functions in Egyptian patients with morbid obesity and the potential predictors of the postoperative thyroid functions.

**Patients and Methods:**

This study included patients undergoing SG at kasr al ainy hospitals. The patients underwent preoperative 3-, 6-, and 12-month postoperative analyses of the thyroid functions and other biochemical markers.

**Results:**

The study included 106 patients who showed significant improvement in thyroid functions at the follow-up assessment. Twelve-month TSH positively correlated with the 12-month measures of LDL and HbA1c. TSH change at 12-month follow-up (TSH) was inversely correlated to 12-month BMI and positively correlated to preoperative TSH and 12-month percentage of total weight loss (TWL%). Univariable linear regression analysis demonstrated that preoperative TSH (*p* < 0.001), 12-month TWL% (*p* = 0.042), 12-month HbA1c (*p* = 0.001), and 12-month LDL (*p* = 0.049) were significant predictors for the 12-month TSH levels. Multivariable analysis showed that only preoperative TSH levels (*p* < 0.001) and 12-month HbA1c levels (*p* = 0.021) could affect the 12-month TSH levels.

**Conclusion:**

The current study supports the evidence of thyroid function improvement after sleeve gastrectomy. This improvement was affected by the amount of weight loss after surgery.

## Introduction

Obesity has shown a recent dramatic increased prevalence during the last 50 years.^1^ It has been established that obesity is a predisposing factor for several comorbidities, including life-threatening conditions.^2^ The ideal choice to treat obesity and have a normal body mass index (BMI) is to follow a healthy lifestyle. However, it usually fails due to non-compliance.^3^ Surgical treatment is the only definite successful treatment for patients with a BMI of 35 kg/m^2^ or higher.^1^

Sleeve gastrectomy (SG) continues to be one of the most popular bariatric techniques all over the world. This is attributable to its reproducibility and high success rates, together with its technical simplicity, the short learning curve, and keeping the physiological integrity of the gastrointestinal tract without a bypass or anastomosis. The short-term follow-up studies have found that SG showed safety with a satisfactory loss of weight and remission of comorbidities.^4–7^

The thyroid hormones are well documented to control dietary intake and energy consumption. Therefore, patients with reduced thyroid functions are likely to develop obesity as well as other metabolic disorders.^8^ On the other hand, adipose tissue influences thyroid axis activity.^9^ Thyroid-stimulating hormone (TSH) frequently shows a slight elevation in patients with obesity.^10,11^ It is uncertain whether this alteration is implicated in the patient’s obesity or, instead, whether the increased fatty mass impacts the thyroid function.^12^

There is an enormous volume of scientific evidence related to SG.^13^ .However, there remains much uncertainty concerning some topics that require more scientific input. The effect of SG on thyroid hormones has been rarely investigated. This study aimed to assess the short-term impact of SG on thyroid functions in Egyptian patients with morbid obesity and the potential predictors of the postoperative thyroid functions.

## Patients and Methods

This is a prospective clinical study that was conducted on prospectively enrolled patients undergoing laparoscopic SG (LSG) at kasr al ainy hospitals during the period from July 2021 to October 2022. The study was commenced after approval by the Research Ethics Committee (REC), code number: MD-174–2021, and per the Helsinki Declaration. Patients were scheduled for bariatric surgery if they were adults with a BMI above 40 kg/m^2^ or 35 kg/m^2^ with comorbid conditions and eligible for surgery under general anesthesia. Patients chosen for LSG according to the institutional protocols were eligible for the current study. The selection of patients for LSG was based on their preferences after a dedicated medical discussion with the surgeon that addressed all surgical options and the benefits and potential side effects of each option. Patients’ recruitment to LSG was performed by an independent surgeon who was not one of the study contributors and was not informed about the study design.

The study patients were subjected to a routine preoperative workup. Blood samples for laboratory assessment were obtained after a 12-h fast. Glycated hemoglobin (HbA1c), low-density lipoprotein (LDL), and high-density lipoprotein (HDL) cholesterol were measured in the study patients. Analysis of serum TSH, free triiodothyronine (fT3), and free thyroxine (fT4) was performed using the electrochemiluminescence immunoassay (ECLIA for use on Elecsys and Cobas e immunoassay analyzers; Roche Diagnostics, Germany). The reference ranges for TSH, fT3, and fT4 levels were 0.270–4.00 µIU/mL, 2.0–4.4 pg/mL, and 0.93–1.7 ng/dL, respectively.

**Fig. 1:**
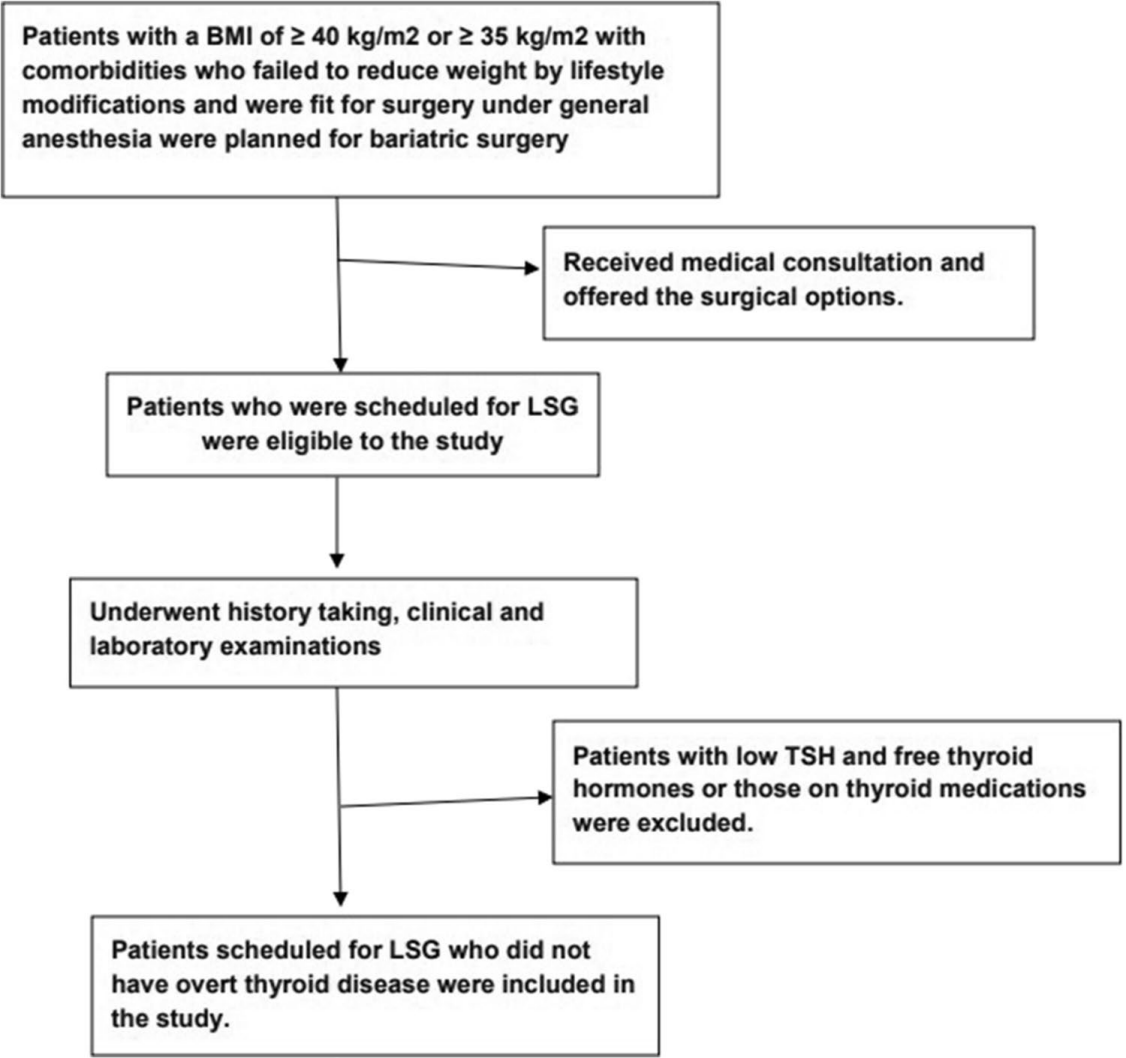
Study flow chart

Patients with overt thyroid disease (patients with abnormal serum TSH as well as free thyroid hormones and those on thyroid medications) were excluded from the study. The study flow chart is shown in Fig. 1. Informed written consent was obtained from the included patients.

### Surgical Technique

The operation was conducted under general anesthesia. The standardized protocol was followed. In summary, pneumoperitoneum was induced, and the procedure was performed via a 5-port technique. The greater curvature vascularity was divided starting from approximately 6 cm proximal to the pylorus until the esophagogastric angle, with the use of a bipolar sealing device (Ligasure). The trans-oral insertion of a 36-Fr calibrated bougie tube and strict positioning adherent to the lesser curve was done. The sleeve line was then stapled with a linear stapler. The methylene blue test was used to exclude leakage, and then, the excised stomach was removed.

### Postoperative Management and Follow-Up

The study patients were motivated to get moving early. A fluid diet was started 2 h after surgery, and a solid diet was introduced gradually during the first 2–3 weeks postoperatively. Patients were prescribed prophylactic anticoagulants for 2 weeks and proton pump inhibitors for 3 months.

Patients’ follow-up visits were scheduled at 3, 6, and 12 months postoperatively. Each visit included a clinical examination, a weight assessment, and an analysis of thyroid function tests. The percentage of total weight loss (%TWL) was calculated as %TWL = [(preoperative BMI − postoperative BMI) / preoperative BMI] × 100.^14^

### The Study Outcomes

The primary outcome of this study was the effect of LSG on the patients’ thyroid functions, and the secondary outcome was the correlation of thyroid functions with the patients’ other investigated parameters as well as the predictors of postoperative TSH levels.

### Statistical Methods

The patients’ data were analyzed using SPSS statistical software (IBM Corp., Armonk, NY, USA) version 26. The normality of numerical data was tested, and then, the student *t*-test was used to compare euthyroid patients to patients with subclinical hypothyroidism, and a repeated ANOVA test was used to compare the patients’ data across the 4 times of measurement, with Bonferroni post hoc correction for the pairwise comparison between every two groups. Different letters were used to define the significantly different pairs. Chi-square and *Z*-score tests for proportion tests were used to compare quantitative variables. The Pearson correlation test was used to assess the potential associations between numerical values. Univariable linear regression analysis was performed to identify the predictors of postoperative TSH levels. The parameters found statistically significant were implemented in a multivariable analysis. Differences were considered statistically significant if the *p*-value was ≤ 0.05.

## Results

Initially, 117 patients were included in the study. Eleven patients were lost during follow-up. The study included the analysis of 106 patients.

The patients’ ages ranged from 31 to 52, with a mean of 42 ± 6.1 years, and the majority were females (94 patients; 88.7%). They had a mean weight of 133.5 ± 24.6 kg and a mean BMI of 50.85 ± 8.3 kg/m^2^. The associated comorbidities were dyslipidemia, hypertension, and diabetes mellitus with a prevalence of 86.8%, 39.6%, and 27.4%, respectively (Table 1).

**Table 1:** Baseline data of the study patients

Age (years)		42 ± 6.1	
Weight (kg)		133.5 ± 24.6	
BMI (kg/m^2^)		50.85 ± 8.3	
		Count	%
Gender	Female	94	88.7
	Male	12	11.3
Comorbidities	Dyslipidemia	92	86.8
	Hypertension	42	39.6
	Diabetes mellitus	29	27.4
Thyroid state	Euthyroid	94	88.7
	Subclinical hypothyroidism	12	11.3

Preoperative laboratory analysis revealed that the mean HbA1c (%) was 6.69 ± 1.17, the mean LDL (mg/dL) was 147.54 ± 21.11, and the mean HDL (mg/dL) was 33.94 ± 4.34. The mean values of TSH (µIU/mL), fT3 (pg/mL), and fT4 (ng/dL) were 2.88 ± 1.09, 2.94 ± 0.49, and 1.2 ± 0.13, and the ranges were 0.7–7.55 µIU/mL, 2.05–4.00 pg/mL, and 0.89–1.7 ng/dL, respectively (Table 2).

**Table 2:** Preoperative and postoperative data of the study patients

	Preoperative	3-month follow-up	6-month follow-up	12-month follow-up	F	p
	Mean ± SD	± mean SD	± mean SD	± mean SD		
Weight (kg)	133.5 ± 24.6^a^	110.11 ± 20.31^b^	93.41 ± 18.49^c^	79.91 ± 17.15^d^	509.85	< 0.001*
BMI (kg/m^2^)	50.85 ± 8.3^a^	41.93 ± 6.59^b^	35.55 ± 6.05^c^	30.41 ± 5.69^d^	571.78	< 0.001*
%TWL	–	17.34 ± 4.94	29.88 ± 6.35	39.99 ± 7.08	–	–
HbA1c (%)	6.69 ± 1.17^a^	5.95 ± 0.47^b^	5.5 ± 0.41^c^	5.4 ± 0.39^c^	53.07	< 0.001*
LDL (mg/dL)	147.54 ± 21.11^a^	128.45 ± 13.36^b^	131 ± 7.95^b^	129.79 ± 7.04^c^	25.61	< 0.001*
HDL (mg/dL)	33.94 ± 4.34^a^	38.15 ± 5.82^b^	42.41 ± 5.18^c^	44.21 ± 2.87^d^	125.97	< 0.001*
TSH (µIU/mL)	2.88 ± 1.09^a^	2.21 ± 0.83^b^	1.82 ± 0.88^c^	1.36 ± 0.53^d^	94.71	< 0.001*
fT3 (pg/mL)	2.94 ± 0.49	2.89 ± 0.43	2.88 ± 0.49	2.87 ± 0.31	0.88	0.46
fT4 (ng/dL)	1.2 ± 0.13^a^	1.17 ± 0.14^b^	1.13 ± 0.19^c^	1.08 ± 0.15^d^	30.62	< 0.001*

*F*, *F* value of the repeated measure ANOVA test; *, statistically significant; different letters indicate a significant pair

The postoperative follow-up data are shown in Table 2. The mean values of TWL% were 17.34 ± 4.94, 29.88 ± 6.35, and 40 ± 7.1 at 3, 6, and 12 months postoperatively, respectively. There was an overall statistically significant reduction in the BMI, HbA1c, LDL, and TSH levels (*p* < 0.001) and a statistically significant increase in fT4 and HDL levels (*p* < 0.001), while there was no statistically significant difference in the fT3 levels (*p* = 0.455). Table 3 shows the change in the studied parameters at the end of the follow-up period.

**Table 3:** Changes in the studied parameters at the end of follow-up period

	Mean ± SD
∆ BMI (kg/m^2^)	20.45 ± 5.62
∆ HbA1c (%)	1.28 ± 1.22
∆ LDL (mg/dL)	17.76 ± 21.3
∆ HDL (mg/dL)	− 10.27 ± 5.52
∆ TSH (µIU/mL)	1.52 ± 0.96
∆ fT3 (pg/mL)	− 0.09 ± 0.54
∆ fT4 (ng/dL)	1.32 ± 0.22

The preoperative TSH levels showed a significant positive correlation with the 12-month TSH (*r* = 0.476, *p* < 0.001). The TSH values at the 12-month follow-up were significantly correlated with the 12-month measures of LDL (*r* = 0.192, *p* = 0.049) and HbA1c (*r* = 0.312, *p* = 0.001). The change in TSH at 12-month follow-up (∆ TSH) negatively correlated with the 12-month BMI (*r* =  − 0.191, *p* = 0.049) and positively correlated with the preoperative TSH (*r* = 0.874, *p* < 0.001) and 12-month TWL% (*r* = 0.327, *p* = 0.001).

The preoperative fT3 significantly correlated with ∆ fT3 (*r* = 0.822, *p* < 0.001), and the preoperative fT4 significantly correlated with ∆ fT4 (*r* = 0.823, *p* < 0.001) and 12-month fT4 (*r* = 0.525, *p* < 0.001).

Comparing patients with subclinical hypothyroidism (*n* = 12) to euthyroid patients (*n* = 94) revealed a statistically significant difference in 12-month TSH (*p* = 0.002), 12-month TWL% (*p* = 0.048), preoperative HbA1c levels (*p* = 0.034), diabetes prevalence (*p* = 0.01), and 12-month LDL (*p* = 0.004) (Table 4).

**Table 4:** Comparison between euthyroid patients and patients with subclinical hypothyroidism

		Euthyroid patients (*n* = 94)		Patients with subclinical hypothyroidism (*n* = 12)		*t*	*p*
		Mean ± SD		Mean ± SD			
Age (years)		41.99 ± 5.94		42.77 ± 6.67		0.46	0.65
Weight (kg)	Preoperative	138 ± 18.41		132.93 ± 25.36		0.67	0.5
	12-month follow-up	76.67 ± 11.13		80.32 ± 17.84		0.69	0.49
BMI (kg/m^2^)	Preoperative	51.35 ± 7.79		50.79 ± 8.4		0.22	0.83
	12-month follow-up	28.78 ± 4		30.61 ± 5.81		1.05	0.3
%TWL		43.55 ± 5.95		39.53 ± 7.11		2.15	0.048*
HbA1c (%)	Preoperative	6.26 ± 0.87		6.83 ± 0.43		2.2	0.034*
	12-month follow-up	5.4 ± 0.39		5.39 ± 1.23		0.13	0.9
LDL (mg/dL)	Preoperative	147.47 ± 21.81		148.14 ± 15.24		0.1	0.92
	12-month follow-up	129.08 ± 6.78		135.29 ± 6.87		2.98	0.004*
HDL (mg/dL)	Preoperative	33.9 ± 4.41		34.28 ± 3.88		0.28	0.78
	12-month follow-up	44.27 ± 3		43.8 ± 1.62		0.53	0.9
12-month TSH (µIU/mL)		1.3 ± 0.48		1.81 ± 0.72		3.4	0.002*
fT3 (pg/mL)	Preoperative	2.88 ± 0.58		2.95 ± 0.48		0.43	0.67
	12-month follow-up	2.82 ± 0.29		2.9 ± 0.48		0.83	0.4
fT4 (ng/dL)	Preoperative	1.2 ± 0.13		1.23 ± 0.14		0.77	0.44
	12-month follow-up	1.08 ± 0.14		1.13 ± 0.19		1.21	0.23
		Count	%	Count	%	χ^2^	*p*
Gender	Female	83	88.3	11	91.7	0.12	0.73
	Male	11	11.7	1	8.3		
		Count	%	Count	%	*Z*	*p*
Comorbidity	Dyslipidemia	82	70	10	14	0.38	0.7
	Hypertension	35	1	7	1	1.41	0.16
	Diabetes mellitus	21	21	2	2	2.56	0.01*

*t*, *t* value of the student *t*-test; χ^2^, Chi-square test; *Z*, *Z* test for proportion; *, statistically significant

Univariable linear regression analysis demonstrated that preoperative TSH (*p* < 0.001), 12-month TWL% (*p* = 0.042), 12-month HbA1c (*p* = 0.001), and 12-month LDL (*p* = 0.049) were significant predictors for the 12-month TSH levels. Implementing these factors in a multivariable analysis showed that only preoperative TSH levels (*p* < 0.001) and 12-month HbA1c levels (*p* = 0.021) could affect the 12-month TSH levels (Table 5).

**Table 5:** Univariable and multivariable linear regression analyses for the prediction of TSH levels at the end of follow-up

	Univariable		Multivariable	
	*p*	Standardized coefficient beta	*p*	Standardized coefficient beta
Preoperative BMI	0.92	0.01		
12-month BMI	0.27	− 0.11		
Preoperative TSH	< 0.001*	0.48	< 0.001*	0.4
Preoperative fT3	0.59	− 0.05		
Preoperative fT4	0.72	− 0.04		
Sex	0.19	− 0.13		
12-month TWL%	0.042*	0.18	0.64	0.04
Age	0.92	0.01		
Preoperative HbA1c	0.13	− 0.15		
12-month HbA1c	0.001*	0.31	0.021*	2.35
Preoperative LDL	0.78	0.03		
12-month LDL	0.049*	0.19	0.18	1.35
Preoperative HDL	0.4	− 0.08		
12-month HDL	0.86	− 0.02		

^*^, statistically significant at *p* ≤ 0.05

## Discussion

Obesity is associated with secondary alterations in various hormones. These alterations include changes in hormone levels and the body’s responsiveness. The interpretation of such alterations in obesity and whether they are clinically relevant is challenging. The adiposity-accompanied thyroid dysfunction and restoring normal function after bariatric surgery remain unclear. The effectiveness of SG has gone beyond being just a weight loss procedure to being documented as a surgical solution for a panel of metabolic disorders.^15^

Few studies investigated the effect of SG on thyroid function.^16–25^ Only two of them were prospective studies,^21,24^ and the remaining were retrospective analyses of patients’ data. The evidence obtained from these studies is still inconsistent. Moreover, no data exist on this association in Egyptian patients with obesity.

In this study, despite normally ranged preoperative fT3 and fT4 levels, the TSH levels ranged from 0.7 to 7.55 µIU/mL denoting a state of subclinical hypothyroidism in some patients with above-normal TSH levels. Our findings are supported by the declaration of the European Society of Endocrinology Clinical Practice Guideline that subclinical primary hypothyroidism is one of the common endocrine disorders in patients with obesity. It is recommended that all patients with obesity should be screened for thyroid function alteration.^26^

Similar findings were reported by Juiz‐Valina et al.^27^, Valdes et al.^10^, and Rotondi et al.^11^ who reported increased TSH levels in patients with obesity. Despite the wide debate over whether the slightly elevated TSH levels are implicated in obesity development or whether obesity itself is the cause of hypothyroidism via activating the hypothalamus-pituitary-thyroid (HPT) axis with subsequent TSH elevation, the latter theory was supported by the recent report of Wang et al.^33^ that was based on genome-wide association studies. The authors found that genetically-driven thyrotropin could not affect BMI.

In variance with other studies,^10,28^ the present work did not find a significant correlation between the preoperative TSH levels and the baseline BMI. This is likely due to all our patients having severe obesity (class 3), while the studies that found this correlation investigated both normal-weight and obese subjects. In line with this finding, the studies of Chen et al. and Moulin de Moraes et al. did not find an association between BMI and TSH levels in patients with severe obesity who were scheduled for bariatric surgery.^16,29^

In this study, a significant gradual reduction of the TSH levels and elevation of fT4 levels was shown at the postoperative follow-up visits indicating the restoration of normal thyroid functions along with the reduction of BMI. This was further confirmed by the positive correlation found between the postoperative TSH and TWL%, the positive correlation between ∆ TSH and TWL%, and the negative correlation between ∆ TSH and the postoperative BMI. In agreement with our study, Chen et al.^16^, Aykota and Atabey,^20^ and Koca et al.^22^ found significant improvement in the TSH and fT4 levels after SG, and the earlier study of Ruiz-Tovar et al. reported a positive correlation between TSH levels and 12-month weight loss.^23^ The studies of Yang et al.^19^, Zhu et al.^25^, and Bawahab et al.^21^ found this significant improvement in the TSH levels only. This variation may be related to the difference in baseline measures or other confounders that may alter thyroid functions.

There is still a lack of understanding of the mechanisms that are plausibly explaining the TSH reduction after bariatric surgery. Among the assumed mechanisms, it was reported that the reduction of leptin levels (an adipose tissue-released hormone) after bariatric surgery may be accountable for the reduced activation of the HPT axis with a resultant decrease in the TSH levels.^30,31^ In addition, weight loss decreases the expression of TSH receptors and hence improves the TSH resistance.^32^

Other than gastric volume restriction as a mechanism of post-SG weight loss, the mechanisms include alterations in some gut hormones, such as reduced ghrelin, the hunger-stimulating hormone.^33^ This reduction is expected to a large extent due to the surgery-related gastric fundus resection. There has been a suggestion that ghrelin could stimulate the release of TSH from pituitary thyrotropic cells.^34^ Then, its reduction would be associated with less activation of the TSH release. Such a proposal could give SG a superiority over Roux-en-Y gastric bypass (RYGB and gastric banding) in the ghrelin reduction-mediated thyroid function restoration effect since the latter procedures do not comprise the resection of the gastric fundus. Further randomized controlled trials investigating this issue will be more conclusive.

The predictors of bariatric surgery-related weight loss are still largely variable among studies. Scarce evidence is available concerning the relationship between baseline thyroid function and weight loss after bariatric surgery. The current study demonstrated a significantly lower rate of weight loss in the patients with subclinical hypothyroidism compared to the euthyroid patients. Then, we assume that higher TSH levels lead to resistance to weight loss. This is likely attributable to the effect of hypothyroidism on the metabolic rate. This also might be mediated by the higher HbA1c levels and diabetes mellitus prevalence in patients with subclinical hypothyroidism since diabetes status was described as one of the postbariatric surgery weight loss predictors.^35^ We believe that this finding could be clinically relevant since a cautious pharmacologic modulation in this group of patients could make them gain more benefit from the intervention. Thus, patients scheduled for bariatric surgery should be screened for subclinical hypothyroidism and offered the appropriate cautious treatment to aid in a proper weight loss outcome and attain the best benefits of the surgery. These recommendations were presented to our department’s policymakers to consider adopting screening for subclinical hypothyroidism in patients scheduled for bariatric surgery and forming a multidisciplinary team with the endocrinology and internal medicine physicians to provide treatment and follow-up protocols for patients found to have subclinical hypothyroidism. Further studies assessing whether a similar outcome would encounter patients undergoing bypass surgery and whether such precautionary protocols would affect the patients’ outcomes were planned.

It is worth noting that the current study found an association between TSH status and LDL and HbA1c levels. Thyroid hormones are well documented to be a mainstay in lipid metabolism.^36^ Hypothyroidism, even if subclinical, is described as being related to the status of dyslipidemia, particularly elevated LDL.^37,38^ As for HbA1c, the study of Ruiz-Tovar et al. showed similar findings.^23^ Diabetes mellitus was reported to be associated with thyroid hormone alteration.^39–41^

## Strength and Limitations

The present study is one of a few prospective studies assessing the impact of SG on thyroid functions, and it is the first study on the Egyptian population. However, the study is limited by the short-term follow-up of the study patients and the small number of patients with subclinical hypothyroidism.

## Conclusion

The current study supports the evidence of thyroid function improvement after sleeve gastrectomy. This improvement was affected by the amount of weight loss after surgery.

## Data Availability

Data is available upon request.
